# Production of Bacteriophages by Listeria Cells Entrapped in Organic Polymers

**DOI:** 10.3390/v10060324

**Published:** 2018-06-13

**Authors:** Brigitte Roy, Cécile Philippe, Martin J. Loessner, Jacques Goulet, Sylvain Moineau

**Affiliations:** 1 Département de Biochimie, de Microbiologie et de Bio-Informatique, Faculté des Sciences et de Génie, Université Laval, Québec, QC G1V OA6, Canada; brigitte.roy.12@ulaval.ca (B.R.); cecile.philippe.1@ulaval.ca (C.P.); 2 Département des Sciences des Aliments, Faculté des Sciences de L’agriculture et de L’alimentation, Université Laval, Québec, QC G1V OA6, Canada; Jacques.Goulet@fsaa.ulaval.ca; 3 Félix d’Hérelle Reference Center for Bacterial Viruses and GREB, Faculté de Médecine Dentaire, Université Laval, Québec, QC G1V OA6, Canada; 4 ETH Zurich, Institute of Food, Nutrition and Health, Schmelzbergstrasse, 7CH-8092 Zürich, Switzerland; martin.loessner@ethz.ch

**Keywords:** *Listeria ivanovii*, bacteriophages, alginate, production, disinfection, phagodisinfection

## Abstract

Applications for bacteriophages as antimicrobial agents are increasing. The industrial use of these bacterial viruses requires the production of large amounts of suitable strictly lytic phages, particularly for food and agricultural applications. This work describes a new approach for phage production. Phages H387 (*Siphoviridae*) and A511 (*Myoviridae*) were propagated separately using *Listeria ivanovii* host cells immobilised in alginate beads. The same batch of alginate beads could be used for four successive and efficient phage productions. This technique enables the production of large volumes of high-titer phage lysates in continuous or semi-continuous (fed-batch) cultures.

## 1. Introduction

*Listeria monocytogenes* is responsible for fatal cases of listeriosis in humans via contaminated food products [1]. This bacterial species is ubiquitous in nature and can contaminate the food processing line at any critical point. The increasing resistance of these pathogens to disinfectants under certain conditions requires the use of higher concentrations of chemical products [2]. Furthermore, bacteria exposed to disinfectants may be more likely to develop antibiotic resistance [3,4]. Despite strict regulatory policies, the occurrence of *L. monocytogenes* still has detrimental consequences for the food industry.

The search for alternatives to overcome these challenges has rekindled interest for bacterial viruses (bacteriophages) in agriculture [5], aquaculture [6], food safety [7], and even in infectious diseases [8,9]. The use of strictly lytic (i.e., virulent) phages infecting *Listeria* as biosanitisers represents an ecological alternative that could reduce the use of chemical compounds and lower the concentrations of toxic residues in the environment [10]. Specific biodisinfectants consisting of suspensions of phages can provide a natural means to control pathogens in processed foods and on contact surfaces. For example, the virulent phage A511 has a very broad host range against several strains of *Listeria* spp. [11,12,13] and could be included in the formulation of this type of biodisinfectants.

Phage biocontrol of *L. monocytogenes* strains was first introduced in 2006 with the commercial product ListShield, which contained a cocktail of phages applicable to various foods. Another product is Phageguard Listex P100, which also aimed to reduce *L. monocytogenes* in a range of food products [14,15,16,17,18]. Moreover, it has been demonstrated that different virulent phages can reduce the *L. monocytogenes* population after adhesion to stainless steel or polypropylene surfaces, and a synergistic effect has been observed by combining phages with quaternary ammonium [19,20,21].

However, even if virulent phages have shown great potential for killing pathogenic or opportunistic food-borne bacteria [22], their production on a large scale often remains challenging. Phage production still involves traditional methods using tubes or Erlenmeyer flasks, or it is done in bioreactors as a batch process [23]. High phage titers can be obtained [24,25], but batch processes require significant manpower and non-operational periods of time that may be limiting [26].

Attempts have been made to overcome the disadvantages of the batch process with continuous phage production. Studies involving chemostats [25,27] have been conducted, as well as two-stage continuous processes or cellstat [25,28,29,30], which consists of culturing bacteria, separately, in the first stage to feed to a second stage when phages are produced. Although chemostat allows the cultivation of microorganisms at a physiological steady state [31], the bacterial culture may be less genetically stable, as mutations can occur [32]. Cellstat is recognised as a phage production system for strictly lytic phages [33] that avoids direct phage exposure and pressure but requires the use of two different bioreactors [34].

With the aim of reducing production time and costs, we investigated here a different phage production procedure that employs host bacteria entrapped in a porous gel matrix. Alginate gel was selected for the matrix because of its low cost and widespread use in a range of applications in medicine, pharmacy, biotechnology, and the food industry [35,36]. An alginate matrix with entrapped bacterial cells can be produced in a single-step process and has virtually no impact on the viability of the cells. Alginate can also form a gel in the presence of divalent cations, such as calcium, which are also often necessary as co-factors for phage multiplication [37,38].

Entrapped cells will still grow because nutrients diffuse through the gel matrix [39], and while microcolonies spread deeper in the beads, the bacterial density has been shown to be higher near to or at the surface of the beads [40,41,42]. Such a growth pattern leads to bacterial cell release in the medium by micro-fracture events in the matrix. Only bacterial cells released from the gel become infected and contribute to the propagation of virulent phages. Those cells remaining in the gel have been shown to be protected from the phages, as the bacterial viruses do not migrate into the beads because of their size [43,44,45]. Protein diffusion through the matrix is highly reduced when molecular weight is above 150 kDa [46].

Relevant advantages of using entrapped cells to produce strictly lytic phages are that the phage lysate can be easily recovered and the alginate beads can be reused for successive phage propagations. Multiple phage lytic cycles can also be favoured, because this protective system prevents the rapid decline of the phage-sensitive host population. This process also provides an opportunity to produce phages in continuous or semi-continuous (fed-batch) cultures. Taken together, the use of calcium alginate immobilised cells (in spheres or fibers) to produce phages is a bi-phasic technique that controls the bacterial population and preserves the integrity of the cells, as long as they remain entrapped in the matrix.

## 2. Materials and Methods

### 2.1. Bacteria, Phages, and Media

*Listeria ivanovii* WSLC 3009 and the broad-host-range virulent myovirus A511 [47] were obtained from the Institut für Mikrobiologie, ZIEL Institute for Food and Health, Technische Universität München (Germany). The siphovirus H387 [48,49] was obtained from the Félix d’Hérelle Reference Center for Bacterial Viruses (www.phage.ulaval.ca) of the Université Laval (Québec, Canada). Bacterial strains were grown in Trypticase soy broth (TSB) or plated on Trypticase soy agar (TSA) at 30 °C. Phage titration was done using the double-layer plating technique [50] on TSA. Phage stocks (>1 × 10^8^ Plaque Forming Unit (PFU) mL^−1^) were stored at 4 °C prior to use.

### 2.2. Alginate Gels and Cell Immobilisation by Entrapment

A 2–4% (*w*/*v*) aqueous solution of sodium alginate was prepared by suspending the polymer in distilled water. Solutions were sterilised by autoclaving (121 °C, 15 min). *L. ivanovii* cells were harvested by centrifugation (8000 rpm, 10 min) and resuspended in sterile TSB (3 mL). The cell suspensions were then mixed with sterile alginate [51]. Beads were formed by the dropwise addition of the alginate-cell mixtures into sterile CaCl_2_ (200 mM) using a syringe and a 20 Gauge (G) needle. The cell-containing beads, 2 to 3 mm in diameter, were allowed to solidify for 1 to 2 h before CaCl_2_ was replaced by fresh TSB containing 0.5 mM CaCl_2_ to maintain the integrity of the alginate beads.

### 2.3. Morphology of Cells Immobilised in Beads

Alginate beads were observed by scanning electron microscopy (SEM) to visualise entrapped *Listeria* cells. The alginate beads were cut in half, and the specimens were fixed by immersion in glutaraldehyde (2.5% *v*/*v*) in 0.1 M sterile cacodylate buffer (pH 7.0) for 4 h. The samples were washed twice in 0.1 M sterile cacodylate for 20 min. Post-fixation was done in osmium tetroxide (2% *v*/*v*) in sterile cacodylate buffer for 30 min at 30 °C, and dehydration was completed using CO_2_ in a critical point dryer (Model 3000 CPD, Bio-Rad, Mississauga, ON, Canada). The samples were mounted on stubs and covered with 15 nm of gold using a sputter coater (Emscope, Bio-Rad). A Nanolab LE 2100 (Vickers Instruments, Bausch and Lomb, Nepean, ON, Canada) scanning electron microscope operating at 15 hV was used to examine the bead surfaces.

### 2.4. Phage Adsorption

A set of alginate beads was made as described above but omitting the bacterial cells. Ten grams of pure alginate beads was transferred into TSB. Aliquots of phage suspensions (0.1 and 1 mL) were added and incubated at 30 °C for 12 h. The adsorption of phages on alginate beads was monitored by determining phage titers every 4 h. Two independent experiments were performed.

### 2.5. Biomass Concentration

To estimate the population of immobilised bacteria, 1 mL of alginate beads was dissolved in 9.0 mL of Na^+^ citrate (50 mM), a sequestrant for Ca^++^. The number of viable cells in the dissolved alginate gel was determined by direct plating on TSA for two independent experiments.

### 2.6. Phage Production

#### 2.6.1. Free Cells

TSB (100 mL) was inoculated (5%) with an overnight culture of *L. ivanovii* 3009 from the Weihenstephan *Listeria* collection (WSLC) and grown to an optical density at 600 nm (OD_600_) of 0.5–0.8. Phages were added at multiplicities of infection (MOIs) of 0.1 (1:10) and 1 (1:1), and the mixture was incubated for 16 h at 30 °C. Phage titers were measured every 4 h for two independent experiments.

#### 2.6.2. Immobilised Cells Used for Single and Successive Phage Propagations

Beads containing entrapped microorganisms were transferred at least 2–4 times into prewarmed (30 °C) fresh TSB before phage production. Ten grams of beads containing *L. ivanovii* cells was added to 100 mL of TSB (OD_600_ of 0.5–0.8), and phage suspensions (at MOIs of 0.1 and 1) were added to the cultures. The flasks were incubated at 30 °C for 16 h, and phage titers were also determined as described above for two independent experiments. Between each successive production, the beads were stored overnight at 4 °C in sterile 2% (*w*/*v*) CaCl_2_. The beads were then washed twice with sterile 2% CaCl_2_ and reactivated as described above before each production.

### 2.7. Statistical Analysis

Mean and standard deviation values were calculated using Microsoft Excel (Microsoft, Redmond, WA, USA).

## 3. Results and Discussion

### 3.1. Morphological Observations

**Figure 1:**
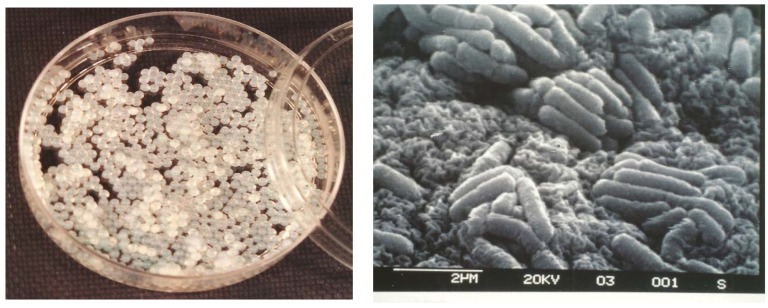
Observation of entrapped alginate bacteria. (**Left**) Visual appearance of alginate beads containing *Listeria ivanovii* WSLC 3009 (10^8^ Colony Forming Unit (CFU) mL^−1^) in a Petri dish. (**Right**) Scanning electron micrograph of *L. ivanovii* WSLC 3009 immobilised in alginate beads (×10,000).

For the efficient use of alginate microbeads, morphological characteristics such as size and shape are important [52]. The produced alginate beads had proper sphericality and were typically 2 to 3 mm in size (Figure 1). Scanning electron micrographs of entrapped *Listeria* revealed no major changes in cell morphology (Figure 1). The mechanical constraints of the polymer did not seem to interfere with cell growth.

### 3.2. Phage Adsorption

Phage adsorption onto the gel matrix is a parameter that may impact the overall performance of the production system. The electrostatic adsorption of phages onto polymer beads could decrease the number and infectivity of phage particles in the medium. For this reason, the organic material selected for phage production should be tested for ionic attraction of viral particles. No decreases in the titers of phages A511 and H387 were observed in the medium after contact with the alginate beads. These results suggest that no major ionic interactions exist between the organic polymer and the phages.

### 3.3. Biomass Concentration

**Figure 2:**
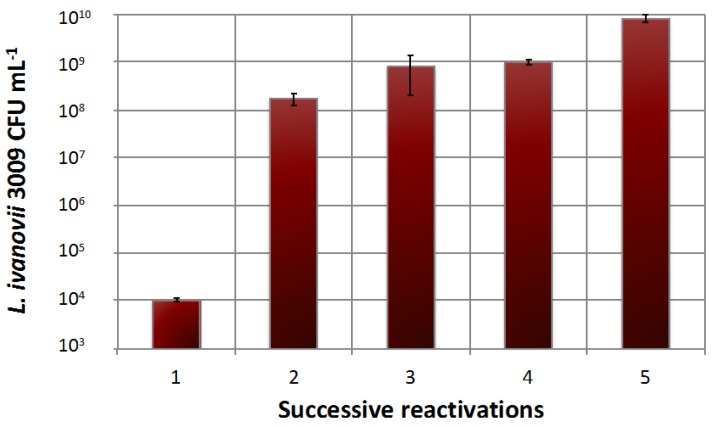
Transfers of *Listeria ivanovii* WSLC 3009 immobilised cells in fresh Trypticase soy broth (TSB) medium. Each reactivation was followed by an interval of 12 h. Bacterial concentrations were measured after 12 h of growth at 30 °C. Mean values were calculated from two independent experiments, and error bars correspond to standard deviations.

The concentration of entrapped cells in alginate beads has been studied for several types of bacteria [39,51,53,54]. Alginate is non-toxic to most living cells [55] and provides protection against external stresses such as temperature, pH, and toxic molecules. Figure 2 shows that three successive transfers (reactivations) of entrapped *L. ivanovii* cells in fresh TSB could raise the bacterial cell concentration inside the gel to almost 1 × 10^9^ cells mL^−1^, while five transfers increased the bacterial counts to almost 10^10^ cells mL^−1^. Because the number of bacteria released into a medium is related to, among other parameters, the saturation level of the cells in the alginate structure, the yield of phage production will likely be influenced by the concentration of bacteria in the beads and at the bead surface.

### 3.4. Phage Production

#### 3.4.1. Free Cells

**Figure 3:**
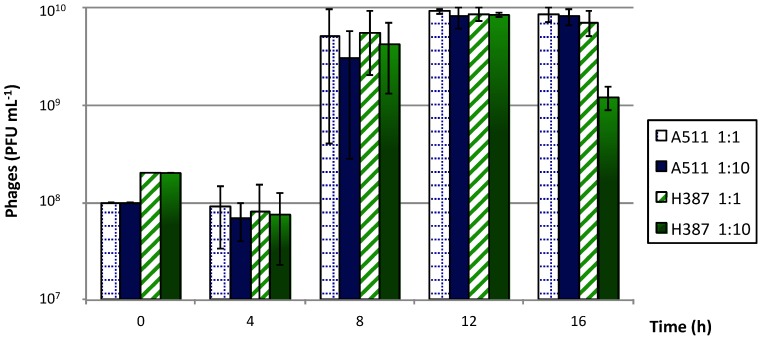
Production of phages A511 and H387 on *Listeria ivanovii* WSLC 3009 in liquid medium, using multiplicities of infection (MOIs) of 1 and 0.1. Phage counts were measured every 4 h. Mean values were calculated from two independent experiments, and error bars correspond to standard deviations.

Phage productions in liquid medium were performed with both phages individually (Figure 3). All phage productions were characterized by a lag phase for the first 4 h, followed by a sharp increase in phage titers at 8 h. Maximal phage titers were close to 10^10^ PFU mL^−1^ of medium after 12 h. Very small variations in phage titers were observed at different MOIs. After 16 h, the titer of phage H387 decreased when using a 1:10 ratio. It is unclear at this time what caused this decrease in the phage titer, but it could have been due to phage adsorption to cell debris.

#### 3.4.2. Immobilised Cells Used for Single and Successive Phage Propagations

**Figure 4:**
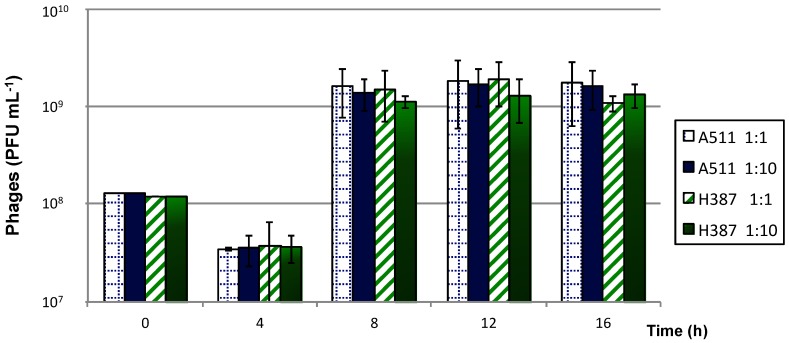
Production of phages A511 and H387 on *Listeria ivanovii* WSLC 3009 immobilised in alginate beads, using 1 and 0.1 multiplicities of infection (MOIs). Phage titers were measured every 4 h. Mean values were calculated from two independent experiments, and error bars correspond to standard deviations.

Microorganisms immobilised in polymers produce concentrated host bacteria that can be more easily and rapidly manipulated than free cells. Phage production using gel-entrapped host cells was compared to that of free cells in the same culture medium and under the same growing conditions. The highest production of virulent *Listeria* phages A511 and H387 was obtained after 12 h using a MOI of 1 (Figure 4). The maximum phage titers achieved using entrapped cells were slightly lower than for free cells. Some phage productions reached their maximum titer after 8 h of incubation, which was faster than for the free cells.

**Figure 5:**
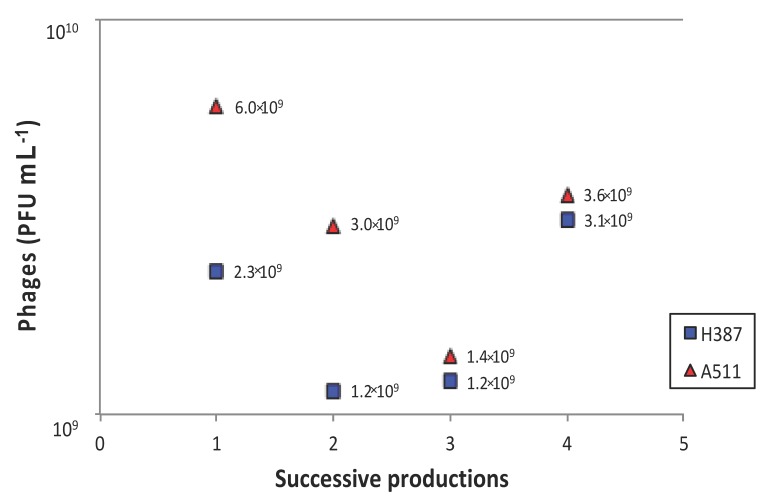
Successive productions of the two phages, A511 and H387, on *Listeria ivanovii* WSLC 3009 using a multiplicity of infection (MOI) of 1. Aliquots were withdrawn after 10 h of incubation. Mean values were calculated from two independent experiments.

Two advantages of using gel-entrapped cells to produce virulent phages are that phage particles can be easily recovered by draining the culture medium (followed by centrifugation and filtration) and that phage propagation can be immediately resumed or pursued after a short or prolonged storage period. The same alginate beads with immobilised *L. ivanovii* cells were used for four successive phage productions. In all cases, phage titers were maintained at over 10^9^ PFU mL^−1^ after the four productions (Figure 5). In general, the final phage titers of the virulent phage A511 were higher than for phage H387.

It has been shown previously that phages infecting some lactic acid bacteria cannot penetrate calcium alginate gels [43,44]. Because *Listeria* phages are the same size as dairy phages and even larger in the case of A511 [56,57], bacterial cells are well protected from phage infection as long as they remain entrapped in the gel. It is likely that this physical constraint, protecting the integrity of the bacterial population, allows the gel beads to be reused for successive phage production in new media. This advantage cannot be provided by free-cell amplification. Only small molecules can diffuse through the alginate matrix [43]. *L. ivanovii* cells entrapped in alginate beads are, therefore, protected against phages as well as against contamination by other bacteria. The production of phages after infection of the host bacteria likely only takes place on the beads’ surface and in the medium after the cells have been released from the matrix. In fact, we noticed that the structure of the alginate gel was rather loose and easily broken up at the periphery of the beads, where cells usually most actively grow. These cells were likely released into the medium and infected by phages.

While the process described here still requires optimisation, the gel entrapment of cells to produce specific phages offers the potential for the large-scale and rapid production of phages. Successive phage productions have shown that entrapped cells can be reused for at least four propagation cycles. Although the viral titer of lysate produced with entrapped cells was nearly 10-fold reduced compared to that of free-cell production, successive productions with the same beads should be globally seen as an interesting advantage. Continuous phage production using entrapped cells could be enhanced and applied to a large variety of phages.
